# Exome sequencing of fetal anomaly syndromes: novel phenotype–genotype discoveries

**DOI:** 10.1038/s41431-018-0324-y

**Published:** 2019-01-24

**Authors:** Nicole Meier, Elisabeth Bruder, Olav Lapaire, Irene Hoesli, Anjeung Kang, Jürgen Hench, Sylvia Hoeller, Julie De Geyter, Peter Miny, Karl Heinimann, Rabih Chaoui, Sevgi Tercanli, Isabel Filges

**Affiliations:** 1 Medical Genetics, Institute of Medical Genetics and Pathology, University Hospital Basel, Basel, Switzerland; 2 Department of Clinical Research, University Hospital Basel, Basel, Switzerland; 3 University of Basel, Basel, Switzerland; 4 Pathology, Institute of Medical Genetics and Pathology, University Hospital Basel, Basel, Switzerland; 5 Department of Obstetrics and Gynecology, University Hospital Basel, Basel, Switzerland; 6 Centre for Prenatal Ultrasound, Freie Strasse, Basel, Switzerland; 7 Centre for Prenatal Diagnosis, Friedrichstrasse, Berlin, Germany

**Keywords:** Clinical genetics, Clinical genetics, Next-generation sequencing

## Abstract

The monogenic etiology of most severe fetal anomaly syndromes is poorly understood. Our objective was to use exome sequencing (ES) to increase our knowledge on causal variants and novel candidate genes associated with specific fetal phenotypes. We employed ES in a cohort of 19 families with one or more fetuses presenting with a distinctive anomaly pattern and/or phenotype recurrence at increased risk for lethal outcomes. Candidate variants were identified in 12 families (63%); in 6 of them a definite diagnosis was achieved including known or novel variants in recognized disease genes (*MKS1, OTX2, FGFR2*, and *RYR1)* and variants in novel disease genes describing new fetal phenotypes (*CENPF, KIF14*). We identified variants likely causal after clinical and functional review (*SMAD3, KIF4A*, and *PIGW*) and propose novel candidate genes (*PTK7, DNHD1*, and *TTC28*) for early human developmental disease supported by functional and cross-species phenotyping evidence. We describe rare and novel fetal anomaly syndromes and highlight the diagnostic utility of ES, but also its contribution to discovery. The diagnostic yield of the future application of prenatal ES will depend on our ability to increase our knowledge on the specific phenotype–genotype correlations during fetal development.

## Introduction

Birth defects are the leading cause of perinatal lethality in industrialized countries [1]. With the advance of high-resolution ultrasound fetal structural anomalies are now detected increasingly early during pregnancy, raising questions about diagnosis, etiology, prognosis, and recurrence risk for parents and health care providers, particularly in the presence of more than one fetal anomaly likely indicating a congenital malformation syndrome. Prenatal high-resolution chromosomal microarray analysis will allow a diagnosis of causal copy number variants in up to additional 10% of pregnancies after the exclusion of the frequent aneuploidies, but 80–90% of families remain without definite diagnosis. Targeted molecular testing may be indicated if a specific monogenic entity is suspected based on clinical signs. The monogenic etiology of phenotypes observed postnatally has been successfully investigated by using genome-wide sequencing technologies [2]. Exome or genome approaches have now become available in routine clinical genetics services for the diagnosis of patients with developmental disorders. Only recently, however, the delineation of fetal anomaly phenotypes received increasing attention including the discussion of introducing exome sequencing (ES) in standard prenatal care. Best et al. [3] discussed its promises and pitfalls including the review of 31 studies with series of five or more fetuses and diagnostic rates varying between 6.2% and 80%. Approaches in such studies are highly heterogeneous ranging from prospective prenatal ES to the study of selected cases with severe fetal anomalies after termination or stillbirth [4]. In general, the diagnostic yield seems to be higher in fetuses with multiple congenital anomalies and in selected series with detailed clinical genetics review [3]. However, a significant proportion of anomaly phenotypes encountered during the antenatal developmental stages may be specific to fetal life since they will lead to embryonic, fetal or perinatal lethality and will have escaped etiological research and clinical delineation so far. They may also represent an incomplete or severe allelic presentation of a phenotype described to occur postnatally, and the diagnosis therefore can remain unrecognized at this stage of development. Filges and Friedman [5] stressed the value of applying genomic sequencing to examine such rare extreme phenotypes despite the challenges to expect when interpreting the clinical significance of variants and proving their causality. Based on these considerations we explored the clinical and molecular diagnosis using ES in a series of 19 families with one or more fetuses with severe structural anomalies. We aimed at identifying causal as well as candidate variants, showing the utility of ES for diagnosis but also discovery, and sought to contribute to the further delineation and etiology of phenotype presentations of known and novel multiple congenital anomaly syndromes in fetal stages intending to increase the yield of future prenatal diagnostic ES.

## Patients and methods

The study was approved by the Ethics Commission Northwest Switzerland (EKNZ 2014-174). After written informed consent for participation was obtained from the parents, we prospectively recruited 19 families—26 fetuses (after fetal or perinatal death or from terminated pregnancies) and one child—that presented with severe anomalies of unexplained etiology initially identified by ultrasound scanning. Families were included in the study if (i) the fetus showed a pattern of two or more anomalies associated with a high risk for fetal or perinatal lethality that suggested a genetic disorder or (ii) there was familial recurrence of the fetal anomaly phenotype and if (iii) there were detailed clinical fetal ultrasound and/or autopsy data available and (iv) high-resolution chromosomal microarray did not show a causal chromosomal anomaly or copy number variant. Families were recruited through the Medical Genetics Clinic at the University Hospital Basel, Switzerland. The fetal phenotypes were reviewed by experienced clinical geneticists, fetal and neuropathologists, and maternal-fetal-medicine specialists. Autopsy was performed in affected fetuses of 18 families.

### Exome sequencing (ES) and variant prioritization

Genomic DNA was used from previous prenatal specimen extracted from chorionic villus sampling, amniocentesis or extracted from fresh frozen or formalin fixed paraffin embedded (FFPE) fetal tissue. Parental DNA was extracted from whole-blood samples. ES was performed in familial trios (or quattros, when available after recurrence). Library preparation (Agilent SureSelect^XT^ Library Prep Kit) and exome capture using the Agilent SureSelect^XT^ Human All Exon V6 (Agilent, Santa Clara, CA) was followed by paired-end read sequencing (2 × 100 bp read length) on a HiSeq 2500 or HiSeq 4000 platform (Illumina, San Diego, CA) with an average coverage of ×100. Quality estimation of the sequence reads was performed by generating quality control statistics with FastQC (http://www.bioinformatics.bbsrc.ac.uk/projects/fastqc). Illumina CASAVA (1.8.2) was used to demultiplex the sequencing reads. Adapter trimming was performed with Skewer (Version 0.2.2). Sequencing reads were mapped and aligned to the reference genome sequence (hg19) with Burrows-Wheeler Aligner (BWA-mem Version 0.7.2). Alignments with a Phred quality score below 30 for the entire read and potential PCR duplicates were discarded using samtools (Version 0.1.18). To increase sensitivity, variant calling was performed with samtools and varscan (Version 2.3.5). Variants with a coverage ≤10X and not supported by at least 4 reads (20%) were discarded. Familial segregation of variants was performed to identify de novo, autosomal recessive and X-linked inheritance. Variants were filtered on a heterozygous population frequency (GMAF) of <5% in control databases (dbSNP142, 1000G, gnomAD), and absence of homozygosity in healthy individuals (ExAC) and classified as known or novel according to their presence or absence in curated databases. Variants were prioritized according to their potential to disrupt protein function using standard prediction tools (SIFT, Provean, Polyphen2, Mutationtaster, Human Splicing Finder v.3.0), amino acid conservation assessment across species (PhyloP, PhastCons) and the American College of Medical Genetics and Genomics (ACMG) variant classification guidelines [6]. In addition, we applied the HOPE protein prediction program (http://www.cmbi.ru.nl/hope/) which combines structural information including the 3D structure of the protein, if available, to analyze the specific mutational effect on the protein structure. Putative candidate variants were visually reanalyzed in each family using the Integrative Genomics Viewer (http://software.broadinstitute.org/software/igv/) and confirmed by Sanger sequencing (primer sequences are available on request). Genotype–phenotype correlations were considered critical for identification of the causal relationship between a candidate variant and anomaly phenotype. Existing information was compiled from medical literature and databases (Pubmed, OMIM, ClinVar, Decipher) and related protein networks and signaling pathways in embryonic development (Reactome, Uniprot, Ingenuity pathway analysis (Qiagen)). We systematically interrogated zebrafish, mouse, and drosophila phenotype databases (www.zfin.org; www.informatics.jax.org; www.mousephenotype.org; www.flybase.org) using cross-species phenotype comparison for validation of candidate genes. GeneMatcher (genematcher.org) was queried for similar cases. We used the HGVS nomenclature Version 15.11 to describe variant effects (https://varnomen.hgvs.org/). All variants identified were submitted to the LOVD database (https://databases.lovd.nl/shared/individuals 00181103-09, 00181141, 00181143-49).

### Functional studies

In 3 fetuses, FFPE tissue from affected organs was used against age-matched control fetal FFPE tissue for reverse transcription analysis of the candidate gene to assess the presence or absence of the transcript. Control tissue was obtained from fetuses without structural anomalies confirmed by autopsy after intrauterine fetal death likely due to asphyxia. RNA from FFPE tissue specimens was isolated with the RecoverAll™ Total Nucleic Acid Isolation Kit for FFPE (Ambion, Thermo Fisher Scientific, Waltham, Massachusetts). Reverse transcription (RT) of mRNA was performed with the High-Capacity cDNA Reverse Transcription Kit (Applied Biosystems, Waltham, MA, USA). 18S RNA primers served as quality control. In 2 fetuses, additional qPCR was used to confirm the lower concentration of the transcript. qPCR was performed with the SYBR Green Master Mix (Applied Biosystems, Waltham, MA, USA). Expression levels of the transcripts were normalized to *GUSB* expression.

## Results

Phenotypes and causal or candidate variants, if identified, are summarized in Table 1. Supplementary Table S1 shows the unsolved cases. The frequency of congenital anomalies in different organ systems is displayed in Supplementary Table S2. We identified variants that would cause or potentially explain the fetal anomaly phenotype in 12 out of 19 families (63%). A definite diagnosis was achieved in 50% of those (6 families), while in the remaining 6 families variants had to be classified as variants of unknown significance according to the ACMG guidelines [6]. Variants in 12 different genes were identified (*RYR1, MKS1, FGFR2, PIGW, CENPF, KIF14, SMAD3, KIF4A, PTK7, DNHD1, TTC28*, and *OTX2*). Variants that were classified to affect or likely affect function were detected in 8 different genes (*RYR1, MKS1, FGFR2, PIGW, CENPF*, *KIF14, SMAD3*, and *OTX2*). The variants identified are categorized into the following groups: (1) a priori diagnostic, since they were known or novel in known disease genes explaining the fetal anomaly phenotype, (2) confirmed to be disease-associated by the identification of additional families and/or functional analysis, and thus led to the description of a new disease gene and related phenotype, (3) likely disease-associated by clinical and functional review in (a) a known disease gene or (b) a new candidate gene.

**Table 1:** Fetal phenotypes and disease-associated and candidate variants identified

Affected individual	Gestational age and sex	Phenotype	Gene	Variants	Type of variant	Inheritance	Variant classification^a^
Family 1, II.1	14 GA male	Meckel-Gruber syndrome	*MKS1* OMIM: 609883	NM_017777.3: c.[417G>A];[417G>A], p.E139 = MAF: 1.9 × 10^−4^	Splicing affected	Autosomal recessive	Affects function, Pathogenic (II)
Family 2, II.1	Birth male	Agnathia-Otocephaly complex	*OTX2* OMIM: 600037	NM_001270523.1: c.[746delG], p.(G249Vfs*45)	Frameshift	De novo	Affects function, Pathogenic (Ia)
Family 3, II.3	22 + 3 GA male	Corpus callosum agenesis, small brain, mesocardia, syndactylies on hands and feet, Apert syndrome	*FGFR2* OMIM: 176943	NM_000141.3: c.[755C>G], p.(S252W) MAF: 9 × 10^−6^	Missense	De novo	Affects function, Pathogenic (Ia)
Family 4, II.4	24 + 5 GA, male	Hydrops, skeletal and smooth muscle hypoplasia	*RYR1* OMIM: 180901	NG_008866.1(NM_001042723.1): c.[9686-1G>C];[9686-1G>C]	Splice site	Autosomal recessive	Affects function, Pathogenic (Ia)
Family 5, BII-2 Filges et al., 2016	32 + 2 GA female	Microphthalmia, Xiphoid cleft, Hydronephrosis, duodenal atresia and «apple peel» atresia	*CENPF* OMIM: 600236	NM_016343.3: c.[1744G>T];[9280C>T], p.(E582*);(R3094) MAF: 2.5 × 10^−5^	Nonsense	Autosomal recessive	Affects function, Pathogenic (Ia)
BII-3 Filges et al., 2016	22 GA male	Xiphoid cleft, bilateral renal hypoplasia, duodenal atresia type III, jejunal atresia, string of beads appearance	*CENPF* OMIM: 600236	NM_016343.3: c.[1744G>T];[c.9280C>T] p.(E582*);(R3094*) MAF: 2.5 × 10^−5^	Nonsense	Autosomal recessive	Affects function, Pathogenic (Ia)
Family 6, Fetus 1 Filges et al., 2014	21 + 4 GA female	Cerebral hypoplasia, cerebellar hypoplasia, agenesis of occipital lobes, bilateral renal agenesis, ureteral agenesis, uterine hypoplasia	*KIF14* OMIM: 611279	NM_014875.2: c.[1750_1751delGA];[1780A>T], p.(E584Ifs*16);(R594*)	Frameshift, Nonsense	Autosomal recessive	Affects function, Pathogenic (Ia)
Fetus 2 Filges et al., 2014	18 + 5 GA female	Corpus callosum agenesis, cerebral hypoplasia, arhinencephaly, bilateral renal hypoplasia and cystic dysplasia, ureteral hypoplasia, uterine hypoplasia, vaginal atresia	*KIF14* OMIM: 611279	NM_014875.2: c.[1750_1751delGA];[1780A>T], p.(E584Ifs*16);(R594*)	Frameshift, Nonsense	Autosomal recessive	Affects function, Pathogenic (Ia)
Family 7, II.1	20 GA male	Agnathia-Otocephaly complex	*SMAD3* OMIM: 603109	NM_005902.3: c.[860G>A], p.(R287Q)	Missense	De novo	Probably affects function, Likely pathogenic (II)
Family 8, II.1	22 + 3 GA male	Hydrocephalus internus	*KIF4A* OMIM: 300521	NM_012310.4: c.[2096T>A], p.(V699E)	Missense	X-linked recessive	VUS
Family 9, II.1	18 GA male	Dandy Walker malformation, hydronephrosis, genital hypoplasia	*PIGW* OMIM: 610275	NM_178517.3: c.[106A>G];[106A>G], p.(R36G) MAF: 2 × 10^−4^	Missense	Autosomal recessive	Probably affects function, Likely pathogenic (II)
II.2	12 GA female	Dandy Walker malformation, dysplastic kidneys, hydronephrosis, diaphragmatic hernia	*PIGW* OMIM: 610275	NM_178517.3: c.[106A>G];[106A>G], p.(R36G) MAF: 2 × 10^−4^	Missense	Autosomal recessive	Probably affects function, Likely pathogenic (II)
Family 10, II.1	22 GA female	Brain malformations, unilateral anophthalmia, hepatomegaly, bile duct atresia, Mullerian duct agenesis	*PTK7* OMIM: 601890	NM_001270398.1: c.[19G>A];[1238A>G], p.(G7R);(N413S) MAF: 0.007, MAF: 3 × 10^−4^	Missense	Autosomal recessive	VUS
Family 11, II.1	22 + 5 GA female	Hypoplastic left heart, persisting left cardinal and vena cava with estuary in the left atrium, atrial septum defect type II, fused lung lobes, absent extrahepatic bile ducts, intestinal stenosis and pouch	*DNHD1* OMIM: 617277	NM_144666.2: c.[6109A>G];[6109A>G], p.(S2037G) MAF: 0.003548	Missense	Autosomal recessive	VUS
Family 12, II.2	33 + 1 GA male	Duodenal atresia III, jejunal and ileal atresia	*TTC28* OMIM: 615098	NM_001145418.1: c.[3638A>G];[794A>C], p.(D1213G);(K165T)	Missense	Autosomal recessive	VUS

*GA* gestational age in weeks, *MAF* Minor allele frequency (gnomAD), *VUS* variant of unknown significance

^a^Nomenclature and variant classification according to HGVS Version 15.11 (https://varnomen.hgvs.org) and ACMG guidelines [6]

### Known or novel variants in disease genes explaining fetal anomalies

We identified variants that affect or likely affect protein function in *MKS1, OTX2, FGFR2*, and *RYR1*. The homozygous variant in *MKS1* was previously reported to affect splicing [7] and explained a recurrent Meckel-Gruber syndrome-like phenotype in family 1. Variants in *OTX2* are reported to cause agnathia-otocephaly in rare instances [8]. Although novel, the de novo frameshift variant (c.[746delG], p.(G249Vfs*45)) was considered to affect function, causing the typical phenotype of agnathia-otocephaly in family 2. In a fetus with bilateral syndactyly of hands and feet and corpus callosum agenesis (family 3), the molecular diagnosis of Apert syndrome was made by identifying a known pathogenic de novo variant in *FGFR2* [9]. For family 4, we confirmed previously that a novel homozygous splice-site variant in the skeletal ryanodine receptor 1 gene (*RYR1)* causes lethal hydrops and severe skeletal and smooth muscle hypoplasia [10].

### Variants in novel genes confirmed to be causal for new fetal phenotypes

In family 5, previously reported by Filges et al. [11], novel compound heterozygous truncating variants in *CENPF* were identified in a fetus with a suggested clinical diagnosis of Strømme syndrome and a sibling with a severe malformation phenotype reminiscent of a ciliopathy phenotype (c.[1744G>T];[c.9280C>T], p.(E582*);(R3094)). The identification of variants in the same gene in unrelated families and functional zebrafish results confirmed causality and a variable phenotype presentation ranging from viable Strømme syndrome to a lethal fetal ciliopathy [11, 12]. Novel compound heterozygous truncating variants were identified in *KIF14* (c.[1750_1751delGA];[1780A>T], p.(E584Ifs*16);(R594*)) in two fetuses with a ciliary phenotype not compatible with any described syndrome in family 6 [13]. This was the first report of a human phenotype caused by bi-allelic truncating variants in *KIF14*. The authors suggested *KIF14* to be a candidate gene for allelic viable phenotypes including isolated microcephaly, which was now confirmed in several patients harboring disease-associated variants in *KIF14* as well [14].

### Candidate variants—likely disease-associated by clinical and functional review

In 6 families potential candidate variants were identified in genes for which other variants are known to cause a postnatal phenotype that, however, significantly differs from the phenotype observed in the fetus (*SMAD3, KIF4A, PIGW*) or that are currently not known to cause a human phenotype at all (*TTC28, PTK7, DNHD1)*. They were prioritized due to their role in distinct pathways and/or similar phenotypes in animal models.

#### Genes in which novel candidate variants may cause a fetal phenotype different from the postnatal phenotype

In family 7, fetal ultrasound identified agnathia-otocephaly complex with complete absence of the mandible and a submandibular position of the ears, cleft palate, and aortic isthmus stenosis. No variants were identified in *OTX2* and *PRRX1* through ES and additional Sanger sequencing, in which disease causing variants were previously reported to cause the agnathia-otocephaly phenotype [8, 15]. The novel de novo missense variant in *SMAD3* was prioritized (c.[860G>A], p.(R287Q)) out of four remaining candidates (*KRT86, PRICKLE1, MYH3, SMAD3*) since SMAD3, OTX2 and PRRX1 are reported to act in the same signaling pathway implicated in the development of the branchial arches [16]. Variants in *SMAD3* were previously reported to cause autosomal dominant Loeys-Dietz syndrome 3 (OMIM: 613795), a connective tissue disorder, presenting with aortic aneurysm, cardiac anomalies, cleft palate and significant micro-/retrognathia. Proteins of the SMAD family play an important role in transcriptional regulation. The variant we identified is located in the MH2-domain of SMAD3 important for the interaction between the different SMAD proteins (Uniprot). According to the HOPE prediction, the mutated residue may disturb the binding properties of the MH2 domain since it cannot form hydrogen bonds and salt bridges. Further functional studies on chicken embryos to prove causality are ongoing.

Among 3 variants identified in different genes (*OSGIN1, KIF4A, BCAT1*) potentially disease-causing according to prediction algorithms we considered the X-linked *novel* hemizygous missense variant in the *KIF4A* gene (c.[2096T>A], p.(V699E)) likely to be associated with the isolated hydrocephalus in the male fetus of family 8. The variant was also present in the healthy mother, consistent with X-linked recessive inheritance. Variants in *KIF4A* are reported to cause X-linked intellectual disability (OMIM: 300923) [17]. However, KIF4A is a motor protein that translocates PRC1, a cytokinesis protein, to the ends of the spindle microtubules during mitosis [18], regulates the PARP1 activity in brain development and the survival of neurons [19] and is a member of the L1CAM recycling pathway. Variants in *L1CAM* are well known to cause X-linked isolated and syndromic hydrocephalus [20]. The novel variant is in the highly conserved PRC1 interacting domain and is predicted to disrupt interaction. qPCR confirmed a significant reduction of *KIF4A* mRNA in brain tissue of the affected fetus (12%) compared to FFPE brain tissue of an age-matched control (Supplementary Figure S1). The low mRNA levels indicate nonsense-mediated decay (NMD) and a likely loss-of-function mechanism.

In family 9 both fetuses presented with a Dandy-Walker malformation, hydronephrosis, dysplastic kidneys, and genital hypoplasia, and an additional diaphragmatic hernia in one fetus, suggesting a clinical diagnosis of Fryns- or Fryns like syndrome. In total, variants in 6 genes were considered (*ABCA1, AIM1L, CTDSP2, NOP16, RSU1*, and *PIGW*). A homozygous missense variant in *PIGW* (c.[106 A>G];[106 A>G], p.(R36G) that segregated in both fetuses was prioritized because phenotypes caused by variants in *PIGV* and *PIGN* genes of the PIG family show a phenotypic overlap with Fryns syndrome [21] or are described to be causal for the Fryns phenotype [22]. The presence of PIGW is required in the early steps of GPI anchor biosynthesis, and bi-allelic variants in genes encoding components of the GPI anchor biogenesis pathway have been suggested to be a rare cause of variable developmental and malformation phenotypes [23]. So far, variants in *PIGW* were reported to cause a glycosylphosphatidylinositol biosynthesis defect 11 (OMIM 610275) presenting with developmental delay, intellectual disability, and seizures [24]. The variants in patients described are closer to the 3′-end of the gene than the one in the fetuses with an anomaly phenotype we identified. This variant is located in the transmembrane domain and the mutant amino acid is predicted to disturb the transport activity of the protein (HOPE).

#### Novel candidate genes

The structural anomalies of the fetus of family 10, including multiple brain anomalies, spina bifida, unilateral anophthalmia, bile duct atresia, agenesis of the Müllerian ducts, hepatomegaly and bilateral cleft palate was not specific for any previously described congenital anomaly syndrome. From the two genes with potentially disease-associated bi-allelic variants (*MTHFD1L, PTK7*) we selected compound heterozygous missense variants in *PTK7* (c.[19G>A];[c.1238 A>G], p.(G7R);(N413S)) as the best candidates since two PTK7 loss of function mouse models show severe neural tube defects, cystic kidneys, micrognathia, and unilateral anophthalmia [25, 26]. No human phenotype has been described so far, but PTK7 deficiency is suspected to play a role in neural tube defects [27], being a key protein in the embryonic Wnt and PCP signaling pathways. Furthermore, PTK7 regulates the outgrowth of the Wolffian duct [28], which is essential for the formation of the Müllerian duct [29]. One of the detected variants (c.19G>A) is located in a transcript (NM_001270398.1), that is coding for the protein isoform e which has an alternative exon 1 that lacks a signal peptide sequence for localization in the cell membrane. Currently, functional properties of this isoform are investigated. RT-PCR on RNA extracted from liver FFPE tissue of the affected fetus and tissue of an age-matched control showed a significant signal decrease in the affected fetus. qPCR of the same samples confirmed a decrease of mRNA expression to 6% (Supplementary Figure S2). These findings are indicative of NMD of the *PTK7* mRNA due to aberrant splicing, at least in the liver and for this isoform.

In family 11, the fetus had a complex heart defect with a hypoplastic left heart and aorta ascendens, subtotal mitral valve atresia, persistent cardinal vein and atrial septal defect, fused lung lobes, gallbladder agenesis, and reduced intrahepatic bile ducts, incomplete intestinal rotation and a pouch-like extension of the proximal jejunum. The malformation pattern was suggestive of a heterotaxy/ciliopathy phenotype. ES detected potentially disease-associated variants in three different genes (*CACNA1A*, *PLCH2*, *DNHD1*). A homozygous missense variant in *DNHD1* (c.[6109 A>G];[6109 A>G], p.(S2037G)) was prioritized due to its function in the dynein heavy chain, although no consanguinity was reported. Variants in other genes such as *DNAL1* (OMIM: 610062), *DNAI1* (OMIM: 604366) and *DNAH11* (OMIM: 603339), which are coding for dynein compartments, are known to cause situs inversus-like phenotypes. Little is known about the exact function of *DNHD1*. It was reported as a candidate gene for intellectual disability [30]. Family 12 presented with recurrence of intestinal atresia. DNA was only available from one child for trio analysis. ES detected compound heterozygosity in 4 different genes (*CASZ1, PLEKHM2, COL5A1*, and *TTC28*), but only variants in *TTC28* (c.[3638 A>G];[c.794 A>C], p.(D1213G);(K265T)) were predicted disease associated and were highly conserved across species. We prioritized *TTC28* because variants in *TTC7A* (OMIM: 609332), a member of the same gene family, causes autosomal recessive gastrointestinal defects, and variants in *TTC21B* (OMIM: 612014) are reported in human ciliopathies.

## Discussion

The overall primary detection rate of disease-associated variants in known disease genes explaining the fetal phenotype is 21% in our highly selected series of fetuses with structural anomalies. For those families counseling to inform about prognosis and recurrence risk as well as reproductive choices, such as prenatal diagnosis or preimplantation genetic diagnosis, in further pregnancies is available. Other studies report detection rates of 10–21% [4, 31–33], however, inclusion criteria and approaches in all studies including ours are highly heterogeneous and therefore restrict comparability. Our detection rate increases after having ascertained disease-association for the variants in the novel candidate genes *KIF14* and *CENPF* by the investigation of additional unrelated affected patients as well as functional and animal model studies [11, 13]. Whereas clinical diagnoses such as agnathia-otocephaly and Meckel-Gruber syndrome were obvious, the presentation of Apert syndrome was atypical since craniosynostosis, the key clinical sign in the corresponding postnatal phenotype, cannot be appreciated at that early developmental stage and therefore hampered targeted molecular genetic testing. This reverse phenotyping highlights the importance of appreciating fetal phenotypes as a variable of developmental timing. Candidate variants identified in the additional 6 families do not have an immediate clinical impact, but may guide the future search for further affected patients and functional studies for confirmatory evidence. Particularly, the suspected causal relationships between variants in *PIGW* for a Fryns syndrome phenotype, KIF4A as a player in the L1CAM pathway for X-linked hydrocephalus and variants in *SMAD3* which may cause agnathia-otocephaly as the severe end of the Loeys-Dietz spectrum emphasize the importance of clinical and developmental genetics review in addition to formal variant assessment. Similarly, the phenotype of about one-third of the fetuses examined, including family 10, 11, and 12, is reminiscent of some type of ciliopathy compatible with the fundamental role of pathways implied in ciliogenesis and ciliary function in embryonic development. Nine of the twelve conditions identified are likely following autosomal recessive inheritance, which may in part reflect recruiting bias including families with phenotype recurrence. However, embryonically lethal mouse models suggest that autosomal recessive inheritance may play an important role in phenotypes with early lethality. The identification of *KIF14* variants causing a lethal ciliary anomaly pattern highlights the significance of investigating such extreme phenotypes for the primary description of a human disease gene, representing the severe phenotypic spectrum of an allelic viable postnatal disorder. Large-scale projects such as the International Mouse Phenotyping Consortium (IMPC) producing knockout mouse lines will be an important resource to support the characterization of novel human disease genes. In four of the seven families in which we were not able to delineate a compelling candidate gene, fetuses presented with recurrence of an isolated anomaly (Supplementary Table S1). This supports the observation of others as well as previous prenatal microarray studies for CNVs that a monogenic etiology is more likely identified in the presence of multiple anomalies. Besides the multiple limitations in fetal phenotyping and exome analysis, multifactorial or polygenic inheritance or epigenetic mechanisms remain a possible explanation. Much more research is needed to explore the utility of genome-wide sequencing in isolated anomalies, particularly when there is phenotype recurrence, potentially also suggestive of a recessive disorder with incomplete phenotype presentation in the antenatal period. The recruitment of additional unrelated fetuses presenting with the same phenotype is one of the most important recognized challenges in elucidating pathogenicity of candidate variants in the prenatal field despite an arising awareness of the importance to investigate such families. Prenatal phenotypes may be imprecise, and autopsy often not done for various reasons impairing phenotype–genotype comparisons and the collection of a larger number of similarly affected fetuses. Functional analysis of potential candidate variants and the generation of appropriate variant specific animal models demands expertize and significant resources. The current diagnostic yield of prenatal ES, however, may remain limited when the interpretation of variants continues to rely on experiences with postnatal patients exclusively. Recruiting patients in a perinatal setting may raise high parental expectations, and a thorough discussion of the current limitations in the interpretation of private variants is required. However, using ES as a discovery tool integrating the delineation of precise fetal phenotypes we contribute to further understand altered developmental pathways specific to fetal life. Recruitment of additional families and studies of variant effects will be mandatory to confirm causal relationships and understand disease mechanisms. Besides the gain of knowledge on biological mechanisms in early human development, this will allow increasing the utility of prenatal ES in a long-term perspective, and much more families will benefit from diagnostic certainty in this sensitive field.

Supplementary information regarding this article is available at (https://www.nature.com/ejhg/)

## Supplementary information

Supplemental Material
