# Supplementary material for: Exome sequencing of fetal anomaly syndromes: novel phenotype–genotype discoveries
Source: Eur J Hum Genet. 2019 Jan 24;27(5):730–7. doi: 10.1038/s41431-018-0324-y (PMC6461982; doi:10.1038/s41431-018-0324-y)
Supplement: Supplementary file 1 — Supplemental Material [file 41431_2018_324_MOESM1_ESM.docx]

| **Affected Individual** | **Gestational age (weeks) and sex** | **Phenotype** |
| --- | --- | --- |
| **Family 13**,  II.6 | 20 GA female, | Microlissencephaly |
| II.7 | 20 GA female | Microlissencephaly |
| **Family 14**,  II.1 | 2y female, | Pierre-Robin sequence, N. facialis palsy, bilateral cleft palate, dysplastic genitals, |
| II.2 | 2d male | Pierre-Robin sequence, bilateral cleft palate, dysplastic genitals, N.facialis palsy not assessed |
| **Family 15**,  II.1 | 22 GA male | Myelomeningocele S-2 |
| II.2 | 20 GA female | Chiari II malformation, Myelomeningocele L-5 |
| **Family 16**,  II.1 | Birth male | Diaphragmatic hernia, lung hypo- and dysplasia |
| II.2 | Birth male | Hypoplastic left heart, diaphragmatic hernia, lung hypo- and dysplasia |
| **Family 17**, II.1 | 19 GA male | Cystic dysplastic kidney right, agenesis of left kidney |
| **Family 18**, II.2 | 21 GA male | Cystic dysplastic kidneys, malrotation of the intestine, anal atresia |
| II.3 | 31+5 GA (birth), male | Extremely fragile skin, lipomatosis of thigh muscles, hypoplasia of muscles and lungs,  hypoplastic genitals, string of beads-like intestine, liver steatosis and hepatomegaly |
| **Family 19**, II.1 | 32+3 GA female | Hydrops fetalis, hyperplastic heart, bilateral lung hypoplasia with lymphangiectasia, «string of beads» like intestine |

**Supplementary Table S1** Fetal phenotypes without identified candidate gene

GA, gestational age

**Supplementary Table S2** Frequency of malformations in different organ systems

| **Affected organ systems** | **Frequency in the cohort (N=26)** | **Specific findings** |
| --- | --- | --- |
| Genitourinary | 16 (62%) | Hydronephrosis, renal cysts, renal agenesis, dys-, hypoplastic genitals, agenesis of mullerian ducts, uterine hypoplasia, vaginal atresia |
| Central nervous | 15 (58%) | Lissencephaly, Dandy-Walker malformation, corpus callosum agenesis, Chiari II malformation, cerebellar hypoplasia, arhinencephaly, hydrocephalus, meningomyelocele, meningoencephalocele, N.facialis palsy, anophthalmia |
| Respiratory | 10 (38%) | Lung hypoplasia, fused lung lobes, pleural effusion, lymphangiectasia, diaphragmatic hernia |
| Gastrointestinal | 10 (38%) | Hypoplastic esophagus, hypoplastic intestine, malrotation of the gut, string of beads like intestine, duodenal stenosis dolichosigma, megacolon, anal atresia |
| Head | 9 (35%) | Microcephaly, macrocephaly, cleft palate, retrognathia, agnathia, |
| Cardiovascular | 8 (31%) | Hypoplastic myocardium, hypoplastic left heart, aortic-, pulmonary valve stenosis, myocardial hyperplasia, Ebstein anomaly, Azygos continuity |
| Skeletal | 7 (27%) | Kyphoscoliosis, scoliosis, rudimental ribs, ulnardeviation, hexadactyly, syndactyly |
| Hepatobiliary | 6 (23%) | Hepatomegaly, siderosis, steatosis, bile duct atresia, bile duct agenesis |
| Neuromuscular | 5 (19%) | Arthrogryposis, muscle hypoplasia, club foot, |
| Hydrops/edema | 5 (18%) | Hydrops, cystic hygroma, aszites |

**Supplementary Figure S1** Expression of *KIF4A* in brain tissue in the fetus of family 14

**Relative expression of KIF4A mRNA in an age matched control fetus, and the fetus affected by hydrocephalus.**

RNA was isolated from FFPE brain tissue. The expression of *KIF4A* was normalized to *GUSB* expression. The expression in the affected fetus is decreased to 12% compared to the unaffected one.

**Supplementary Figure S2** Expression of *PTK7* in liver tissue in the fetus of family 6

**Relative expression of PTK7 mRNA in an age matched control fetus, and the affected one**

RNA was isolated from FFPE liver tissue. *PTK7* expression was normalized to *GUSB* expression. The expression level of *PTK7* in the affected fetus is 6% compared to the level of the unaffected one**.**
